# The Bar of Isis

**Published:** 1909-02-13

**Authors:** 


### Editor's Letter-Box.

### "THE BAR OF ISIS."

(*To the Editor of* The Hospital.)

Sir,—May I be allowed to remark regarding the review of the book bearing the title above, which appeared in your columns of December 26, that the respect for authority is fortunately giving place to research for the truth of the authority, and unless the truth substantiates the authority, the latter becomes null and void. The authority for the statement that "fertilisation is fermentation" is based upon the well-known and recognised scientific data of Professor Jacques Loeb and other leading biologists on the general chemistry of life phenomena. Thus "the egg is not the bearer of the form of the full-grown animal, but of certain chemical substances, especially of ferments . . . the subsequent physical changes which the material undergoes will be under the influence of the catalytic agencies of both the egg and the spermatozoön." Numberless experiments in artificial parthenogenesis testify to this assertion. The gynæcologist who still regards *vernix caseosa* as a "natural protective and lubricant" is indeed a believer in "old fables," long discarded as untenable by those who will have the truth and nothing but the truth. Bayer's "Modern Researches" in this matter are endorsed by hundreds of medical men and women in this country and America; and so important to the future welfare of the race is considered the promulgation of the various reforms advocated in "The Bar of Isis," that already an international league is being formed for that end whose members hail from all parts of the world, and who are determined that the Law of the Mother shall prevail and in time redeem diseased humanity.

Yours faithfully,

The Author of "The Bar of Isis."

*** The notice referred to was hardly in the nature of a review, and our correspondent does not better her case much. As was pointed out, to hoist up a single conjecture into the position of an absolute law is always mischievous, and a thing avoided in competent summaries of scientific matters. As to the "hundreds of medical men and women" who believe that the *vernix caseosa*, for the routine removal of which every nursing manual gives directions, is a pathological product caused by sexual intercourse in excess of that necessary for procreation, and giving rise to serious infantile complaints—for these ladies and gentlemen (if engaged in much midwifery practice) the time must indeed seem out of joint. But we somehow doubt whether this latest of innumerable "leagues" will do much towards setting it right.—Editor, The Hospital.

